# LSTM-Based VAE-GAN for Time-Series Anomaly Detection

**DOI:** 10.3390/s20133738

**Published:** 2020-07-03

**Authors:** Zijian Niu, Ke Yu, Xiaofei Wu

**Affiliations:** School of Information and Communication Engineering, Beijing University of Posts and Telecommunications, Beijing 100876, China; zijian@bupt.edu.cn (Z.N.); wuxf@bupt.edu.cn (X.W.)

**Keywords:** anomaly detection, VAE-GAN, time series

## Abstract

Time series anomaly detection is widely used to monitor the equipment sates through the data collected in the form of time series. At present, the deep learning method based on generative adversarial networks (GAN) has emerged for time series anomaly detection. However, this method needs to find the best mapping from real-time space to the latent space at the anomaly detection stage, which brings new errors and takes a long time. In this paper, we propose a long short-term memory-based variational autoencoder generation adversarial networks (LSTM-based VAE-GAN) method for time series anomaly detection, which effectively solves the above problems. Our method jointly trains the encoder, the generator and the discriminator to take advantage of the mapping ability of the encoder and the discrimination ability of the discriminator simultaneously. The long short-term memory (LSTM) networks are used as the encoder, the generator and the discriminator. At the anomaly detection stage, anomalies are detected based on reconstruction difference and discrimination results. Experimental results show that the proposed method can quickly and accurately detect anomalies.

## 1. Introduction

In recent years, with the development of the Industrial Internet, industrial big data has become an important research topic. Due to the complicated production process, large number of sensors and high sampling frequency, it is easy for industrial equipment to accumulate a large amount of time series data in a short time [1,2]. Some anomalies occurring in the production process will cause the industrial equipment to shut down. Detecting anomalies early can improve the equipment’s overall equipment effectiveness by implementing early preventive maintenance. The industrial time series data has the characteristics of large scale and week periodicity. Designing an effective anomaly detection method for it is a very valuable subject and is also the work done in this paper.

Anomalies, also referred to as outliers, are defined as observations that deviate so much from the other observations as to arise suspicions that they were generated by different mechanisms [3,4]. Most scholars give the definitions of time series anomalies based on this and the actual application field. Anomaly detection has been studied in a variety of data domains including high-dimensional data, uncertain streaming data, network data, and time series data [5,6,7,8,9,10,11,12]. A significant amount of work has been performed in time series anomaly detection. In the statistics literature, several models were proposed, including autoregressive integrated moving average (ARIMA), cumulative sum statistics (CUSUM), exponentially weighted moving average (EWMA), etc [13,14,15,16]. However, in the face of industrial time series data, traditional time series anomaly detection methods cannot meet the expected requirements in efficiency and accuracy. In the past few years, a large number of unsupervised anomaly detection methods based on deep learning have been developed [17,18]. Many scholars use neural network to learn the unknown relationship in time series data, and then build a prediction model to detect anomalies by the deviation of the predicted value from the actual value at each time point. For example, an anomaly detection method based on LSTM prediction model was modeled on normal time series data, and anomalies were identified by comparing the residual of the predicted value and the true value [19,20,21]. Malhotra et.al use stacked LSTM networks trained on non-anomalous data as a predictor over a number of time steps for anomaly detection in time series [22]. Other prediction models include multilayer perceptron (MLP) predictor and support vector regression. With the development of industrial systems, time series data become more and more complicated. In the industrial production process, the behavior of machines always changes based on usage and external factors that are difficult to capture [23]. Under such circumstances, it is difficult to predict the time series even in a few time steps, resulting in the time series anomaly detection method based on the prediction model being no longer applicable. 

In order to solve such problems, some reconstruction-based models were proposed. Anomaly detection methods based on autoencoder (AE) appeared. An encoder learns a vector representation of the input time-series and the decoder uses this representation to reconstruct the time-series. The method based on AE performs anomaly detection through reconstruction difference [24,25,26,27]. AE is a representative reconstruction approach that is a connected network with an encoder and a decoder. It has also been applied for reconstructing time-series data using a sliding time-window [28]. Subsequently, some time series anomaly detection methods based on variational autoencoder (VAE) were proposed [23]. Unlike an AE, a VAE models the underlying probability distribution of observations using variational inference. At present, a novel time series anomaly detection method based on GAN has been proposed [29]. The LSTM networks are used as the generator and the discriminator to capture the distribution of the time series. However, the method based on GAN needs to find the best mapping from real-time space to the latent space at the anomaly stage. This optimization process of finding the best mapping brings new errors and takes a long time, so that the system cannot provide early warning in time.

In this paper, we propose a LSTM-based VAE-GAN for time series anomaly detection, which effectively solves the above problems. The encoder, the generator and the discriminator are jointly trained to take advantage of the mapping ability of the encoder and the discrimination ability of the discriminator simultaneously. In order to capture time dependence, LSTM networks are used as the encoder, the generator and the discriminator. The model is trained on the normal time series. The encoder maps the input time series to the latent space. The generator reconstructs the input time series. The characteristics of the discriminator make it possible to judge anomalies directly from the input time series [30]. Since the encoder, the generator and the discriminator are jointly trained at the training stage, it is not necessary to calculate the best mapping from real-time space to the latent space at the anomaly detection stage. The time required at the anomaly detection stage is greatly reduced, which can make our model detect anomalies more quickly. At the same time, due to the joint optimization of the various modules of our model, our model can detect anomalies more accurately.

In addition, our model design is inspired by a method applied in images of faces, which combines variational autoencoder with a generative adversarial network and shows that this method outperforms VAEs with element-wise similarity measures in terms of visual fidelity [31,32,33,34].

To summarize, the main contributions of our work are:

- A novel anomaly detection method based on VAE-GAN is proposed to detect anomalies in times series data from sensors.
- Our method jointly trains the encoder, the generator and the discriminator, which takes advantage of the mapping ability of the encoder and the discrimination ability of the discriminator simultaneously.
- The anomaly score consists of the reconstruction difference of the VAE part and the discrimination results of the discriminator, which makes it more able to distinguish anomalies from normal data.

## 2. Materials and Methods

### 2.1. Time Series

A time series is a series of data points indexed in time order. Most commonly, it is a sequence taken at successive equally spaced points in time.

We use two time series datasets in our experiment. They are Yahoo and KPI commonly used for evaluating the performance of time-series anomaly detection. In these datasets, anomaly points are labeled as positive, and normal points are labeled as negative.

**Figure 1:**
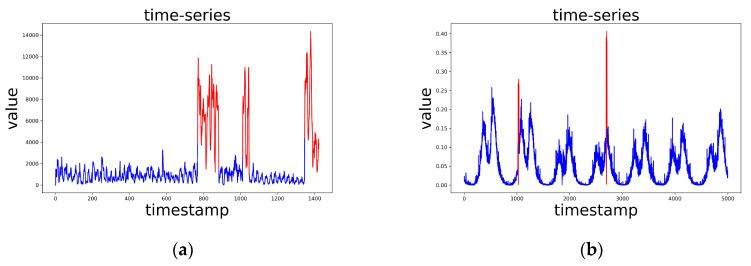
Time series used in our experiment, and the red parts are anomalies. (**a**) An example from Yahoo dataset. (**b**) Time series in KPI dataset.

Yahoo is released by Yahoo Labs. It contains both real and synthetic time series with varying trend, noise and seasonality, representing the metrics of various Yahoo services [35]. The Yahoo dataset has four different parts, the first part A1Benchmark is real data, and the other three parts are synthetic data. The timestamps of the A1Benchmark are replaced by integers with the increment of 1, where each data-point represents 1 hour worth of data. The A1Benchmark has 94866 points in total, 1669 of which are anomalies, and the anomaly rate is 1.76%. We only use the real time series A1Benchmark to evaluate the anomaly detection methods, as shown in Figure 1a.

KPI is released by AIOPS Challenge [36]. It is collected from some Internet Companies, such as Sogo, Tencent, eBay, etc, which reflect the health status of machines (servers, routers, and switches) and quality of Web services. We take the first 10000 points and down sample it every 2 timestamps. After that, we obtain the dataset with 5000 timestamps length, 38 anomaly points, 0.76% anomaly rate, as shown in Figure 1b.

In the data preprocessing, we use the min–max normalization to bring all values in each time series into the range [0,1] and divide each time series into two halves according to the time. Since our model is aimed at learning the distribution of normal data at the model training stage, we remove the anomalies in the data of the first part to get the normal training data. The data in the second part is used for testing. In addition, we divide the time series into training data and testing data into sub-sequences by a sliding window with a size of 10 and a step-size of 3.

### 2.2. LSTM-Based VAE-GAN

**Figure 2:**
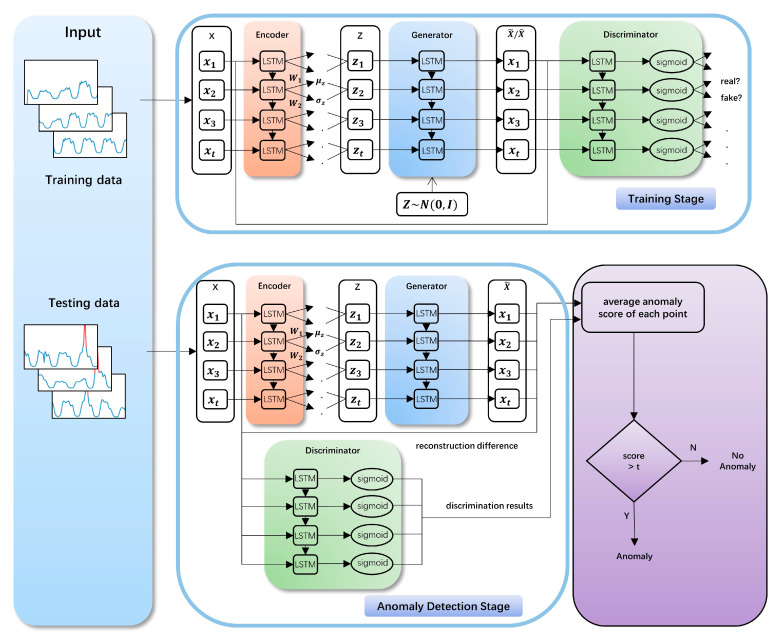
LSTM-based VAE-GAN architecture. $\mu_{z}$ and $\sigma_{z}$ are obtained by the linear transformation of the encoder output. $W_{1}$ and $W_{2}$ are the coefficients of the linear transformation. $z=\mu_{z}+\sigma_{z}\;⊙\;\epsilon$, where ϵ~N(0,I) and ⊙ signify an element-wise product.

This paper presents a LSTM-based VAE-GAN method for time series anomaly detection. The method has two stages, one is the model training stage and the other is the anomaly detection stage. Our model is trained on the normal time series data to learn the distribution of them at the model training stage and calculates the average anomaly score of each point in testing time series data by identifying whether the testing time series data conform to the normal time series data distribution at the anomaly stage. The architecture of LSTM-based VAE-GAN is shown in Figure 2.

Our model is trained on normal data and learns the distribution of normal data. To make the VAE-GAN learn the temporal dependence of time series, we combine the VAE-GAN with LSTM by using LSTM as the encoder, the generator and the discriminator of VAE-GAN. We divide the time series into sub-sequences by a sliding window in a certain step size, which corresponds to the input variables. Each input sample to the encoder is a vector of a certain size denoting the sub-sequence which is encoded to the vector in the latent space. The generator uses the vector in the latent space to generate the vector in the real-time space. The discriminator outputs a vector which denotes whether the vector in the real-time space obeys the distribution of the normal training data.

At the training stage, the encoder approximates the posterior distribution q(z|x) and encodes a data sample x to a latent representation z. The generator reconstructs $\tilde{x}$ by decoding the latent representaion back to data space. As the same time, a random variable $\hat{z}$ is sampled from p(z)=N(0,I), which is a standard normal distribution used for the prior and sent to the generator to generate $\hat{x}$. The LSTM of the discriminator is followed by a full connection with activation function sigmoid. With x, $\tilde{x}$ and $\hat{x}$ as inputs, the discriminator will learn to discriminate data by making $\tilde{x}$, $\hat{x}$ close to 0, and x close to 1.

(1) z~Enc(x)=q(z|x),

(2) $$\tilde{x}\sim Gen\left(z\right)=p\left(x|z\right),$$

For time series, instead of VAE reconstruction error, we use a reconstruction error expressed in the discriminator and obtain better results [34].

The loss of VAE-GAN consists of three parts. For the encoder,

(3) $$L_{enc}=L_{kl}+L_{re},$$

with

(4) $$L_{kl}=KL(q(z|x)||p\left(z\right)),$$

(5) $$L_{re}=-E_{q\left(z|x\right)}\left[\log\left(p\left(Dis^{\prime }\left(x\right)|z\right)\right)\right],$$

where KL is the Kullback–Leibler divergence, $L_{re}$ is the reconstruction of x by maximizing the log-likelihood $\log\left({Dis}^{\prime }\left(x\right)|z\right)$ with sampling from q(z|x), and $Dis^{\text{′}}\left(x\right)$ is the representation of the hidden LSTM in discriminator.

For the generator,

(6) $$L_{gen}=-\log\left(Dis\left(\hat{x}\right)\right)-\log\left(Dis\left(\tilde{x}\right)\right)+L_{re},$$

For the discriminator,

(7) $$L_{dis}=-\log\left(1-Dis\left(\hat{x}\right)\right)-\log\left(1-Dis\left(\tilde{x}\right)\right)-\log\left(Dis\left(x\right)\right),$$

We trained the encoder, the generator and the discriminator in LSTM-based VAE-GAN using Adam optimizers with a 0.001 learning rate.

### 2.3. Anomaly Score

At the anomaly detection stage, the time series for anomaly detection is also divided into sub-sequences by a sliding window in the same step size as the training stage, which are input into the encoder. The encoder maps inputs into the latent space and sends their latent representation to the generator. The generator outputs reconstructed sub-sequences $X_{test}$. The discriminator outputs the possibility of inputs being normal.

The anomaly score utilizes the encoder, the generator, and the discriminator simultaneously trained in the model training phase, which is composed of reconstruction difference and discrimination results. Since anomalies do not conform to the distribution of normal data, their anomaly scores will be relatively high.

(8) $$anomaly_{score}=\left(1-\alpha \right)\left|X_{test}-{\hat{X}}_{test}\right|-\alpha Dis\left(X_{test}\right),$$

Due to the moving window mechanism, some points’ anomaly scores are calculated many times, and some points’ anomaly scores are calculated only once. For each point in time series, the anomaly detector needs to calculate its average anomaly score. 

In addition, we used a small part of the test set containing anomalies to select the optimal threshold. This threshold can accurately distinguish the anomalies in this part, and then generalize to the entire test set.

### 2.4. Anomaly Detection Algorithm

Our method is divided into two stages, namely the model training stage and the anomaly detection stage. After the data preprocessing described above, we can obtain the normal training data and the testing data. The detailed algorithm flow is described in Algorithm 1.
**Algorithm 1.** Anomaly detection algorithm used the LSTM-based VAE-GAN**Input:** training data $X_{train}$, testing data $X_{test}$**Output:** anomaly or no anomaly**At training model stage:**  Initialize Enc, Gen, Dis  In each iteration:    Generate random mini-batch X from training data $X_{train}$     Generate Z from encoder Z=Enc(X)     Generate $\tilde{X}$ from generator $\tilde{X}=Gen\left(Z\right)$     Sample $\hat{Z}$ from prior p(Z)=N(0,I)     Generate $\hat{X}$ from generator $\hat{X}=Enc\left(\hat{Z}\right)$     Update parameters of encoder according to gradient     $\theta_{Enc}\overset{+}{\leftarrow }-{𝛻}_{Enc}\left\{KL\left(q\left(Z|X\right)|p\left(Z\right)\right)-E_{q\left(Z|X\right)}\left[\log\left(p\left({Dis}^{\prime }\left(X\right)|Z\right)\right)\right]\right\}$     
Update parameters of generator according to gradient     
$\theta_{Gen}\overset{+}{\leftarrow }-{𝛻}_{Gen}\left\{-\log\left(Dis\left(\hat{X}\right)\right)-\log\left(Dis\left(\tilde{X}\right)\right)-E_{q\left(Z|X\right)}\left[\log\left(p\left(Dis\prime \left(X\right)|Z\right)\right)\right]\right\}$     Update parameters of discriminator according to gradient     
$\theta_{Dis}\overset{+}{\leftarrow }-{𝛻}_{Dis}\left\{-\log\left(1-Dis\left(\hat{X}\right)\right)-\log\left(1-Dis\left(\tilde{X}\right)\right)-\log\left(Dis\left(X\right)\right)\right\}$**At anomaly detection stage:**  Calculate reconstruction difference: $Re=\left|X_{test}-Gen\left(Enc\left(X_{test}\right)\right)\right|$  Calculate discrimination results: $Dis=Dis\left(X_{test}\right)$  Calculate anomaly score: score=|−αDis+(1−α)Re|  Calculate average anomaly score for each point of time series corresponding to the testing data $X_{test}$  if (score > threshold):    return anomaly  else:    return no anomaly

## 3. Results

### 3.1. Comparision with Other Reconstruction Models in F1 Score

In the LSTM-based VAE-GAN, the LSTM networks for the encoder, the generator and the discriminator have the same size with depth 1 and 60 hidden units. In addition, we set the dimension of latent space as 10.

We use the Precision, Recall and F1 score to evaluate the anomaly detection performance of our model.

(9) $$Precision=\frac{TP}{TP+FP}$$

(10) $$Recall=\frac{TP}{TP+FN}$$

(11) $$F1=\frac{2\times Precision\times Recall}{Precision+Recall}$$

where TP is the number of anomaly points correctly detected, FP is the number of normal points incorrectly identified as anomaly points, and FN is the number of anomaly points incorrectly identified as normal points.

To evaluate the performance of the proposed method, we implemented three baseline methods which are the representative time series anomaly detection methods based on sample reconstruction. They all perform anomaly detection through reconstruction difference.

- LSTM-AE: An anomaly detection method using an LSTM-based autoencoder [28].
- LSTM-VAE: A anomaly detector using a variational autoencoder. Unlike an AE, a VAE models the underlying probability distribution of observations using variational inference. The LSTM networks are used as the encoder and decoder [23].
- MAD-GAN: An anomaly detection method based on Generative Adversarial Networks which uses the LSTM networks as the generator and the discriminator [29].

Table 1 shows the best results of our method LSTM-based VAE-GAN and those representative time series anomaly detection methods based on sample reconstructions. LSTM-AE, LSTM-VAE, and MAD-GAN all use LSTM networks as the basic modules and their basic parameters are the same as those in LSTM-based VAE-GAN. In order to focus on comparing the ability of the model to distinguish between anomalies and normal points, we use the same threshold selection strategy described in this paper for all methods. As shown in Table 1, our method consistently outperforms the other time series anomaly detection methods based on sample reconstruction in F1 score.

### 3.2. Time Spent in the Anomaly Detection Stage

**Figure 3:**
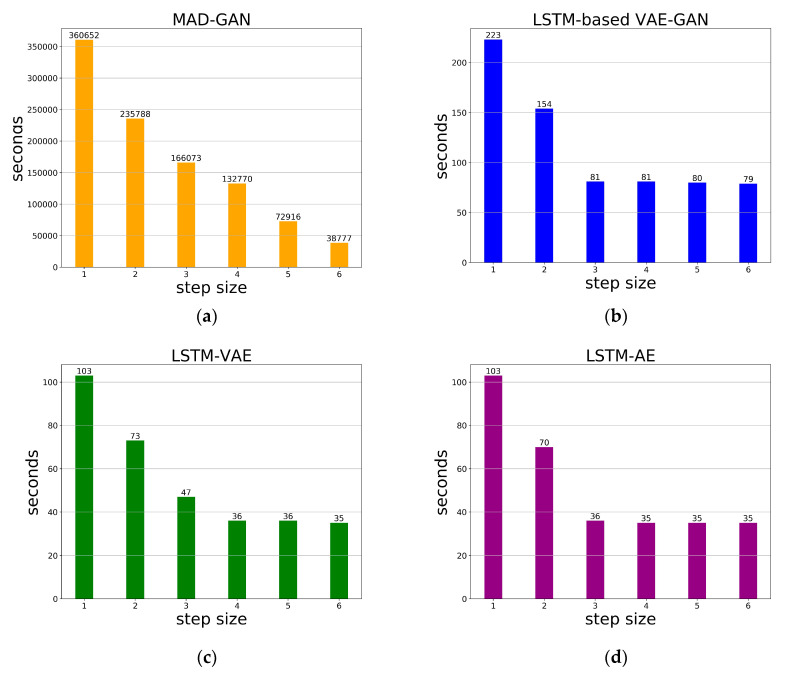
With different step sizes, the time spent by four methods at the anomaly detection stage in Yahoo dataset. (**a**) MAD-GAN, (**b**) LSTM-based VAE-GAN, (**c**) LSTM-VAE, (**d**) LSTM-AE.

Compared with the time series anomaly detection method based on GAN, since LSTM-based VAE-GAN jointly trains the encoder, the generator and the discriminator at the training stage, it does not need to calculate the best mapping from real-time space to the latent space at the anomaly detection stage. The time required at the anomaly detection stage is greatly reduced, which can make the model detect anomalies quicker. We do the time loss experiment on the hardware environment of 2.10 GHz CPU (24 cores, x86 64 architecture), Unbuntu OS and RAM with 128 G. Figure 3 shows the time spent by four methods in Yahoo at the anomaly detection stage, respectively. As Figure 3 shows, during the anomaly detection stage, the time required by our model at each step size is much shorter than the time required by the method based on GAN. Compared with LSTM-AE and LSTM-VAE, LSTM-based VAE-GAN needs to calculate the discrimination results of input, so it takes a little longer than LSTM-AE and LSTM-VAE. In addition, because the number of samples decreases with increasing step size, the time required for both methods decreases as the step size increases.

### 3.3. The Impact of Latent Space’s Dimensions

The latent space representation of our data contains all the important information needed to represent our original data point. This representation must then represent the features of the original data. The representation capability of latent space varies with the dimensions of latent space. We observe the effect of latent space’s dimensions on the performance of the reconstruction-based models in time series anomaly detection. We set the dimensions of latent space to 5, 10, and 15, respectively. Table 2 describes the performance of LSTM-AE, LSTM-VAE, MAD-GAN, and LSTM-based VAE-GAN in different latent space’s dimensions in Yahoo dataset.

### 3.4. Visual Analysis

**Figure 4:**
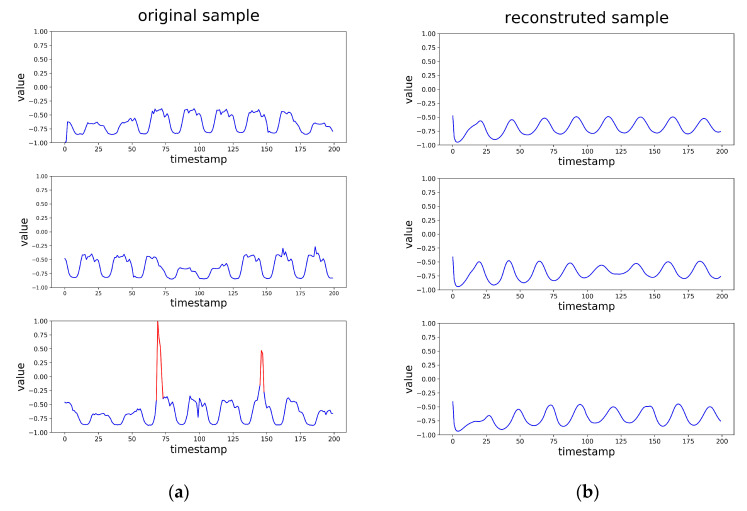
(**a**) The time subsequence needed to detect anomalies, and the red parts are anomalies. (**b**) The reconstructed time subsequence corresponding to the time subsequence in part a.

The LSTM-based VAE-GAN was trained on normal data and learns the distribution of normal data. Since the anomaly samples do not obey the distribution of normal data, the generator cannot reconstruct them well when inputting anomaly samples to the encoder. In order to observe this intuitively, we draw the input time sub-sequences and the reconstructed time sub-sequences in Figure 4. It shows that the normal samples and reconstructed samples of them are roughly the same. When the input sample contains anomaly points as the red part in the figure, the reconstructed sample does not reproduce abnormal points, which provides the possibility for anomaly detection.

**Figure 5:**
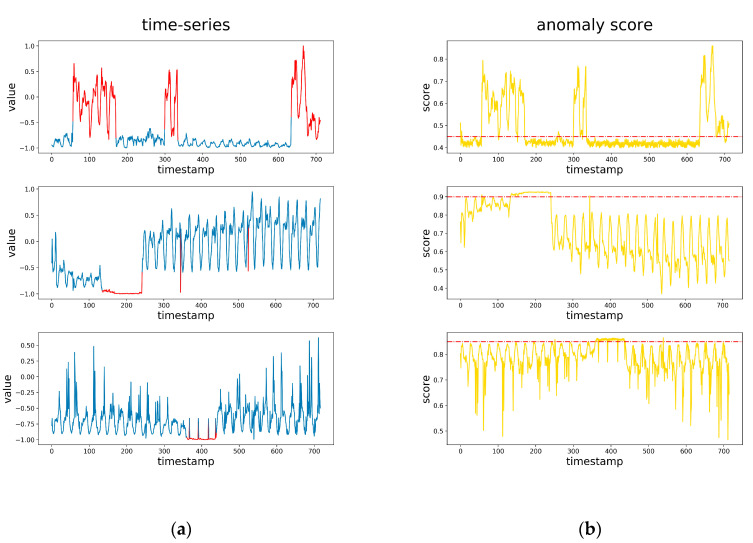
(**a**) The original time series containing anomalies, and the red parts are anomalies. (**b**) The anomaly score corresponding the time series in part a, and the dotted line is the optimal threshold.

Figure 5 shows the anomaly score of the time series, which were outputted by our model. The red dotted line is the optimal threshold. It can be seen that the scores of normal points are mostly below the optimal threshold, and the scores of anomaly points are mostly above the optimal threshold. Since our reconstructed samples are relatively smooth as shown in Figure 4, the reconstruction differences that are part of the anomaly score make the anomaly score curves and the time series shape approximately the same.

## 4. Discussion

In this paper, a LSTM-based VAE-GAN anomaly detection method for time series is proposed. The method is designed to monitor the equipment sates through the data collected in the form of time series.

The time series anomaly detection method based on sample reconstruction can be divided into two stages. One is the model training stage, where the model learns the distribution of normal data. The other is the anomaly detection stage, where the anomaly score of the time series is calculated to identify anomaly. The LSTM-based VAE-GAN jointly trains the encoder, the generator and the discriminator to take advantage of the mapping ability of the encoder and the discriminatory ability of the discriminator simultaneously. The optimization process at the anomaly detection stage is avoided so that anomalies can be detected more quickly and more accurately. In experiments based on Yahoo and KPI time series data, our method has a higher F1 value than several classic sample-reconstruction based time series anomaly detection methods. In the time loss comparison with GAN, our method is shown to spend less time due to avoiding the optimization process at the anomaly detection stage. Due to the moving window mechanisms, some points’ anomaly scores are calculated many times, the others are calculated only once. The accuracy is not influenced by the number of calculations of the anomaly scores at the anomaly detection stage. In fact, the moving window mechanism is not essential in the data preprocessing. It depends on the length of the time series. For increasing the number of subsequences used to train the model at the training stage, we set the step size smaller than subsequence length. If the length of the time series is long enough, the time series can be divided at the same interval.

Although our method can accurately and quickly detect anomalies in time series, there are still some limitations. In our paper, anomalies in time series refer to anomaly points, and the anomaly score module is designed for this background. In some application scenarios where anomalies in time series may be successive anomaly subsequences, anomaly subsequence can be detected if some points in it are detected by the model. A new design of the anomaly score module is needed to meet the application scenarios. 

Our research has room for further development. In the current situation, our method needs to accumulate certain data to adjust the threshold of the anomaly score. The next enhancement of this method is to provide an adaptive threshold adjustment method for quick use.

## Figures and Tables

**Table 1:** Precision, Recall and F1 score of representative time series anomaly detection methods based on sample reconstruction and our method LSTM-based VAE-GAN.

Dataset	Method	Precision	Recall	F1
Yahoo	LSTM-AE	0.4353	0.848	0.5753
	LSTM-VAE	0.8464	0.8516	0.849
	MAD-GAN	0.6007	0.8509	0.7042
	LSTM-based VAE-GAN	0.8752	0.9067	0.8907
KPI	LSTM-AE	0.9474	0.4737	0.6316
	LSTM-VAE	0.76	0.5	0.6032
	MAD-GAN	0.9444	0.4474	0.6071
	LSTM-based VAE-GAN	0.95	0.5	0.6552

**Table 2:** The experiment results in different latent space’s dimensions in Yahoo dataset.

Method	Latent Dim	Precision	Recall	F1
LSTM-AE	5	0.6095	0.7171	0.6589
	10	0.4353	0.848	0.5753
	15	0.4861	0.855	0.6198
LSTM-VAE	5	0.7513	0.872	0.8072
	10	0.8464	0.8516	0.849
	15	0.8281	0.8822	0.8543
MAD-GAN	5	0.6071	0.8434	0.706
	10	0.6007	0.8509	0.7042
	15	0.795	0.887	0.8385
LSTM-based VAE-GAN	5	0.9	0.8577	0.8784
	10	0.8752	0.9067	0.8907
	15	0.8698	0.9054	0.8873

## References

[1] Da Xu L., He W., Li S. (2014). Internet of things in industries: A survey. IEEE Trans. Ind. Inform..

[2] Marjani M., Nasaruddin F., Gani A., Karim A., Hashem I.A.T., Siddiqa A., Yaqoob I. (2017). Big IoT data analytics: Architecture, opportunities, and open research challenges. IEEE Access.

[3] Martí L., Sanchez-Pi N., Molina J.M., Garcia A.C.B. (2015). Anomaly detection based on sensor data in petroleum industry applications. Sensors.

[4] Chandola V., Banerjee A., Kumar V. (2009). Anomaly detection: A survey. Acm J..

[5] Gupta M., Gao J., Aggarwal C.C., Han J., Engineering D. (2014). Outlier detection for temporal data: A survey. IEEE Trans. Knowl. Data Eng..

[6] Pham D.-S., Venkatesh S., Lazarescu M., Budhaditya S.J.D.M., Discovery K. (2014). Anomaly detection in large-scale data stream networks. Data Min. Knowl. Discov..

[7] Aggarwal C.C., Subbian K. Event detection in social streams. Proceedings of the 2012 SIAM International Conference on Data Mining.

[8] Aggarwal C.C., Yu P.S. Outlier detection for high dimensional data. Proceedings of the 2001 ACM SIGMOD International Conference on Management of Data.

[9] Aggarwal C.C., Yu P.S. Outlier detection with uncertain data. Proceedings of the 2008 SIAM International Conference on Data Mining.

[10] Aggarwal C.C. On abnormality detection in spuriously populated data streams. Proceedings of the 2005 Siam International Conference on Data Mining.

[11] Aggarwal C.C., Zhao Y., Philip S.Y. Outlier detection in graph streams. Proceedings of the 2011 IEEE 27th International Conference on Data Engineering.

[12] Gao J., Liang F., Fan W., Wang C., Sun Y., Han J. On community outliers and their efficient detection in information networks. Proceedings of the 16th ACM SIGKDD International Conference on Knowledge Discovery and Data Mining.

[13] Chalapathy R., Chawla S. (2019). Deep learning for anomaly detection: A survey. arXiv.

[14] Pincus R.J.B.J., Barnett V., Lewis T. (1994). Outliers in Statistical Data.

[15] Contreras J., Espinola R., Nogales F.J., Conejo A.J. (2003). ARIMA models to predict next-day electricity prices. IEEE Trans. Power Syst..

[16] Rousseeuw P.J., Leroy A.M. (2005). Robust Regression and Outlier Detection.

[17] Qin Y., Song D., Chen H., Cheng W., Jiang G., Cottrell G. A dual-stage attention-based recurrent neural network for time series prediction. Proceedings of the 26th International Joint Conference on Artificial Intelligence.

[18] Wu X., Shi B., Dong Y., Huang C., Faust L., Chawla N.V. RESTFul: Resolution-Aware Forecasting of Behavioral Time Series Data. Proceedings of the Conference on Information and Knowledge Management.

[19] Bontemps L., McDermott J., Le-Khac N.-A. Collective anomaly detection based on long short-term memory recurrent neural networks. Proceedings of the International Conference on Future Data and Security Engineering.

[20] Hundman K., Constantinou V., Laporte C., Colwell I., Soderstrom T. Detecting spacecraft anomalies using lstms and nonparametric dynamic thresholding. Proceedings of the 24th ACM SIGKDD International Conference on Knowledge Discovery & Data Mining.

[21] Chauhan S., Vig L. Anomaly detection in ECG time signals via deep long short-term memory networks. Proceedings of the 2015 IEEE International Conference on Data Science and Advanced Analytics (DSAA).

[22] Malhotra P., Vig L., Shroff G., Agarwal P. Long short term memory networks for anomaly detection in time series. Proceedings of the 23rd European Symposium on Artificial Neural Networks, Computational Intelligence and Machine Learning.

[23] Park D., Hoshi Y., Kemp C.C. (2018). A multimodal anomaly detector for robot-assisted feeding using an lstm-based variational autoencoder. IEEE Robot. Autom. Lett..

[24] Zhang C., Song D., Chen Y., Feng X., Lumezanu C., Cheng W., Ni J., Zong B., Chen H., Chawla N.V. A deep neural network for unsupervised anomaly detection and diagnosis in multivariate time series data. Proceedings of the AAAI Conference on Artificial Intelligence.

[25] Zong B., Song Q., Min M.R., Cheng W., Lumezanu C., Cho D., Chen H. Deep autoencoding gaussian mixture model for unsupervised anomaly detection. Proceedings of the 6th International Conference on Learning Representations.

[26] Guo Y., Liao W., Wang Q., Yu L., Ji T., Li P. Multidimensional time series anomaly detection: A gru-based gaussian mixture variational autoencoder approach. Proceedings of the Asian Conference on Machine Learning.

[27] Chen R.-Q., Shi G.-H., Zhao W.-L., Liang C.-H. (2019). Sequential VAE-LSTM for Anomaly Detection on Time Series. arXiv.

[28] Malhotra P., Ramakrishnan A., Anand G., Vig L., Agarwal P., Shroff G. (2016). LSTM-based encoder-decoder for multi-sensor anomaly detection. arXiv.

[29] Li D., Chen D., Goh J., Ng S.-K. MAD-GAN: Multivariate Anomaly Detection for Time Series Data with Generative Adversarial Networks. Proceedings of the 28th International Conference on Artificial Neural Networks.

[30] Goodfellow I. (2016). NIPS 2016 tutorial: Generative adversarial networks. arXiv.

[31] Kingma D.P., Welling M. Auto-encoding variational bayes. Proceedings of the 2nd International Conference on Learning Representations.

[32] Rezende D.J., Mohamed S., Wierstra D. Stochastic backpropagation and approximate inference in deep generative models. Proceedings of the 31st International Conference on Machine Learning.

[33] Goodfellow I. On distinguishability criteria for estimating generative models. Proceedings of the 3rd International Conference on Learning Representations.

[34] Larsen A.B.L., Sønderby S.K., Larochelle H., Winther O. Autoencoding beyond pixels using a learned similarity metric. Proceedings of the 33rd International Conference on Machine Learning.

[35] Yahoo Webscope Dataset S5-A Labeled Anomaly Detection Dataset. https://webscope.sandbox.yahoo.com/catalog.php?datatype=s&did=70.

[36] AIOps Challenge. http://iops.ai/competition_detail/?competition_id=5&flag=1.

